# Surviving the storm: exploring the role of natural transformation in nutrition and DNA repair of stressed *Deinococcus radiodurans*

**DOI:** 10.1128/aem.01371-24

**Published:** 2024-12-09

**Authors:** Dhirendra Kumar Sharma, Ishu Soni, Yogendra Singh Rajpurohit

**Affiliations:** 1 Molecular Biology Division, Bhabha Atomic Research Centre 29445 , Mumbai, India; 2 Homi Bhabha National Institute (DAE-Deemed University) 232022 , Mumbai, India; Washington University in St. Louis, St. Louis, Missouri, USA

**Keywords:** natural transformation, *Deinococcus radiodurans*, endonuclease A (EndA), ComEA and ComEC, extracellular DNA (eDNA)

## Abstract

*Deinococcus radiodurans*, a natural transformation (NT)-enabled bacterium renowned for its exceptional radiation resistance, employs unique DNA repair and oxidative stress mitigation mechanisms as a strategic response to DNA damage. This study excavates into the intricate roles of NT machinery in the stressed *D. radiodurans*, focusing on the genes *comEA*, *comEC*, *endA*, *pilT*, and *dprA*, which are instrumental in the uptake and processing of extracellular DNA (eDNA). Our data reveal that NT not only supports the nutritional needs of *D. radiodurans* under stress but also has roles in DNA repair. The study findings establish that NT-specific proteins (ComEA, ComEC, and endonuclease A [EndA]) may contribute to support the nutritional requirements in unstressed and heavily DNA-damaged cells, while DprA contributes differently and in a context-dependent manner to navigating through the DNA damage storm. Thus, this dual functionality of NT-specific genes is proposed to be a contributing factor in the remarkable ability of *D. radiodurans* to survive and thrive in environments characterized by high levels of DNA-damaging agents.

**IMPORTANCE:**

*Deinococcus radiodurans* is a bacterium known for its extraordinary radiation resistance. This study explores the roles of NT machinery in the radiation-resistant bacterium *Deinococcus radiodurans*, focusing on the genes *comEA*, *comEC*, *endA*, *pilT*, and *dprA*. These genes are crucial for the uptake and processing of eDNA and contribute to the bacterium nutritional needs and DNA repair under stress. The findings suggest that the NT-specific proteins ComEA, ComEC, and EndA may help meet the nutritional needs of unstressed and heavily DNA-damaged cells, whereas DprA plays a distinct role that varies, depending on the context in aiding cells to cope with DNA damage. The functionality of NT genes is proposed to enhance *D. radiodurans* survival in environments with high levels of DNA-damaging agents.

## INTRODUCTION

Natural transformation (NT) in bacteria, a form of horizontal gene transfer, is a genetically regulated process that unfolds in an orderly fashion (1). Its primary aim is to acquire extracellular DNA (eDNA) from the environment for various purposes including genome evolution (2–4), DNA repair (5–7), and fulfilling nutritional needs (8, 9). NT is a driving force in bacterial evolution (10–12), yet its effects on genome stability and removal of deleterious genetic elements remain contentious (13–16). Research into NT across different bacterial species highlights its varied effects on adaptive evolution, suggesting a multifaceted role that can be advantageous (16, 17), indifferent (18), or contingent on circumstances (17, 19–21). The direct beneficial impacts of DNA uptake on individual cells could be a nutrient source such as nitrogen, carbon, and nucleotides (10, 12, 21) or a template for DNA repair mechanisms (5–7), with experimental findings corroborating these hypotheses across bacterial diversity. Nonetheless, the acquisition of genetic material from the external environment via NT may carry risks, including the burdens of replication, transcription, and metabolism posed by new genes, as well as potential disruptions to regulatory and protein interaction networks (22). Additionally, the recipient cell faces significant concern regarding the acquisition of genomic parasites, which all self-replicating cell genomes must protect against (23).

*D. radiodurans*, a radioresistant bacterium, and this remarkable radioresistance arise from a synergy of multiple strategies. These include efficient repair of DNA double-strand breaks (DSBs) (24–37), protection of DNA and proteins from oxidation (38–43), antioxidant protection (31, 38, 44–64), novel DNA repair proteins (65–76), cellular signaling constituting serine/threonine kinase and cell division regulation (48, 77–90), a compact nucleoid structure (65, 89, 91, 92), and small RNA-based regulation of DNA repair pathways (93), all working together to ensure cell survival after exposure to extremely high doses of gamma rays, prolonged desiccation, and other DNA-damaging agents. Additionally, *D. radiodurans* maintains its competency of eDNA acquisition throughout the exponential growth phase, experiencing a decline in transforming frequency only during the stationary phase (94). DNA transport machinery is conserved in *D. radiodurans*, and a model for the NT of *D. radiodurans* has been proposed (95). The DNA uptake machinery is similar to that in *Vibrio cholerae*, comprising homologs of pilins PilIV (DR0548 and DR1232), outer membrane channel PilQ (DR0774), ATPases PilB (DR1964) and PilT (DR1963), and pre-pilin peptidase PilD (DR2065). Additionally, it includes a hypothesized inner membrane channel analogous to PilC in *V. cholerae* (96). External DNA traverses the outer membrane via the PilQ channel, facilitated by the DNA-binding protein ComEA (DR1855) or ComEA-like protein (DR0207), which draws the DNA into the periplasm. Subsequently, periplasmic endonuclease A (EndA, DR1600) converts the double-stranded DNA (dsDNA) to single-stranded DNA (ssDNA) by degrading one DNA strand, and only one strand of DNA is translocated into the cytoplasm through the ComEC (DR1854) (inner membrane channel), likely assisted by ComF (DR1389). Once inside, the transforming ssDNA is protected by DNA-binding proteins such as single-stranded DNA-binding (SSB) protein, DdrB, and DprA (DR0120). Differential processing occurs between plasmid and genomic DNA: DprA, bound to genomic DNA, promotes RecA loading onto the genomic DNA, initiating homologous recombination (HR) with the host cell genome, while DdrB or RecO, acting independently of RecA, facilitates enabling of plasmid DNA circularization and establishment through its single-strand annealing (SSA) activity (30, 95, 97). RecO protein serves as a versatile transformation factor, potentially replacing DdrB for plasmid DNA fragment annealing or substituting SSB/DprA for RecA loading onto genomic DNA, aiding in recombination through the RecFOR pathway (95, 98). The experimental validation confirmed the significance of various proteins in NT, revealing that gene mutants of Tfp proteins (*ΔpilQ*, *ΔpilIV*, *ΔpilD*, *ΔpilT*, and *ΔpilB*), along with genes encoding proteins responsible for DNA transfer through the cytoplasmic membrane (*ΔcomEC* and *ΔcomEA ΔcomEC*), exhibited a complete absence of transformation, except for *ΔcomF*, which demonstrated approximately a 2,000-fold reduction in transformation efficiency (95). Moreover, proteins like DprA, DdrB, and RecO, crucial for the terminal stage of NT, displayed redundant roles in the transformation of *D. radiodurans*, as only the double mutants *ΔdprAΔddrB* and *ΔdprAΔrecO* exhibited a total loss of transformation (95, 99).

Together, published reports suggested that Tfp, ComEC-ComEA, and DprA play a crucial role in natural transformation by binding eDNA to transfer through cytoplasmic membrane, safeguarding, and assisting the integration of incoming DNA into the bacterial chromosome, respectively (95). Additionally, we have recently reported on the roles of DprA in ssDNA binding, which prevents degradation by nucleases (63). Furthermore, DprA has been shown to play crucial roles in the differential coordination of DSB repair pathways, contributing to gamma radiation survival in *D. radiodurans* (24). To explore deeper into the specific roles of NT genes (*pilT*, *endA*, *comEA*, *comEC*, and *dprA*) in surviving the onslaught of DNA damage, we assessed the survival and capacity for repairing DSBs in these genes’ mutant background. Our findings establish that NT-specific genes might contribute differently and in a context-dependent manner to navigating through the DNA damage storm. Additionally, our findings indicate that eDNA plays a role in providing nutritional support to stressed cells, yet utilizing eDNA for DNA repair appears to have detrimental or inconclusive consequences.

## RESULTS

### Natural transformation-specific gene mutants showed differential cell survival response to gamma radiation and mitomycin C

**Fig 1:**
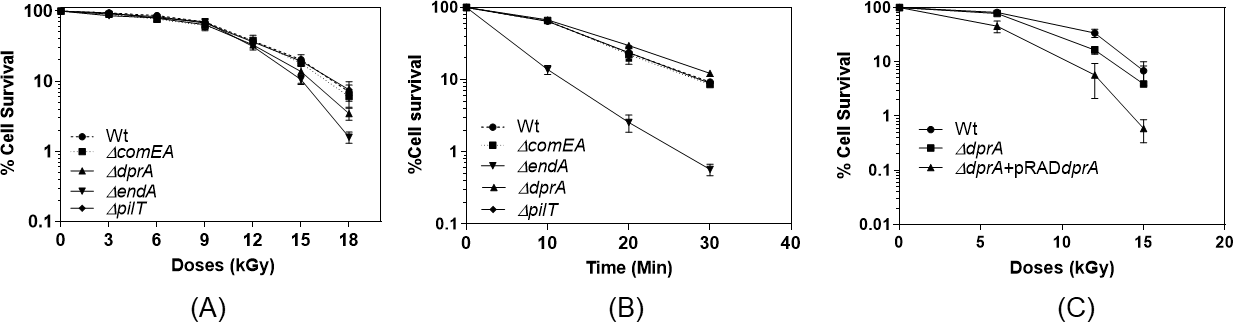
The cell survival of wild-type and natural transformation (NT) mutants of *D. radiodurans* after exposure to gamma radiation and MMC. All data were derived as mean ± SEM of three independent experiments. (**A**) Exponential growth phase cells of wild -ype (-●-), *ΔcomEA* (-■-), *ΔendA* (-▼-), *ΔdprA* (-▲-), and *ΔpilT* (-♦-) mutants were subjected to different doses of gamma radiation, and the percentage of cell survival fraction was plotted as a function of the gamma radiation doses (kGy). (**B**) Exponential growth phase cells of wild-type (-●-), *ΔcomEA* (-■-), *ΔendA* (-▼-), *ΔdprA* (-▲-), and *ΔpilT* (-♦-) mutants were exposed to MMC (10 µg/mL) doses ranging from 0 to 30 minutes. After dilution, cells were plated on TGY (1% tryptone, 0.1% glucose, and 0.5% yeast extract) medium and incubated at 32°C for 48 hours. The percentage of cell survival fraction was plotted as a function of the MMC treatment time. (**C**) Exponential growth phase cells of wild-type (-●-), *ΔdprA* (-■-), and *ΔdprA* mutant harboring pRAD*dprA* (-▲-) were subjected to different doses of gamma radiation, and the percentage of cell survival fraction was plotted as a function of the gamma radiation doses (kGy).

NT integrates eDNA into bacterial genomes through a multistep process involving NT-specific genes and proteins like ComEA, ComEC, ComF, EndA, and DprA (100–102). Importantly, translocated DNA is being protected from cytoplasmic nuclease degradation by proteins like DprA and SSB (103). To understand the roles of NT-specific genes in cell survival under stress in *D. radiodurans*, mutants of NT genes (*ΔpilT*, *ΔendA*, *ΔcomEA*, and *ΔdprA*) were generated, and their cell survival was monitored following exposure to gamma radiation and mitomycin C (MMC). The *ΔcomEA* and *ΔpilT* gene mutants exhibited cell survival similar to wild-type when exposed to various doses of gamma radiation (Fig. 1A). On the other hand, the *ΔendA* mutant showed decrease in the cell survival by one-log cycle at 18-kGy gamma radiation dose, while *ΔdprA* showed decrease in cell survival at higher doses of gamma radiation (>12-kGy) (Fig. 1A). MMC had a profound effect on the *ΔendA* mutant (~1.6-log reduction) and a modest effect on the *ΔdprA* (~0.2-log reduction) in cell survival, while the survival of the *ΔcomEA* and *ΔpilT* mutants was nearly equal to that of wild-type *D. radiodurans* when treated with MMC (10 µg/mL) for 30 minutes (Fig. 1B). The transcomplementation of *dprA* gene from pRAD*dprA* plasmid (a constitutive expression) in *ΔdprA* mutant gives a dominant negative effect on cell survival in the *ΔdprA* mutant strain overexpressing DprA at the tested gamma radiation doses (6, 12, and 15-kGy) (Fig. 1C). Additionally, the transexpression of the *endA* gene in the *ΔendA* mutant was unsuccessful, as the *ΔendA* mutant could not be transformed with the pRAD*endA* plasmid.

**Fig 2:**
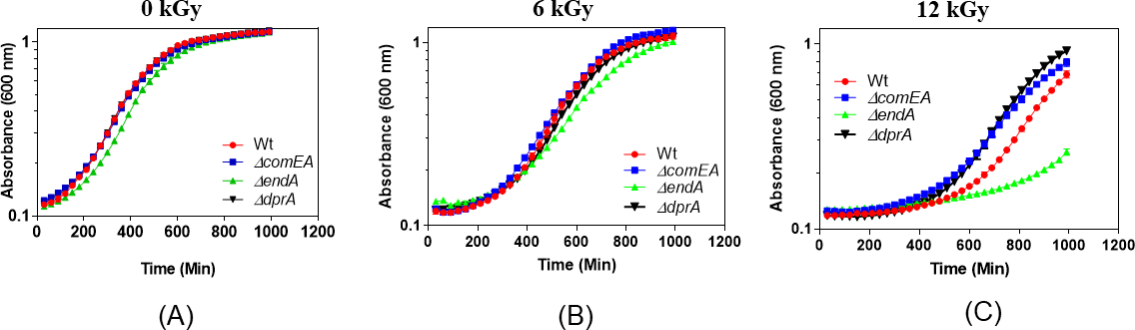
Cell viability and growth curve of wild-type and its NT mutants after exposure to gamma radiation. Optical density at 600 nm was measured continuously using a microtiter-based density reader, and the growth medium (TGY) served as a blank for the entire incubation period. The obtained data were normalized with the blank optical density. (**A**) Growth of normal, untreated cells. (**B**) Cells treated with 6-kGy of gamma radiation. (**C**) Cells treated with a dose of 12-kGy of gamma radiation. Data are represented here as the mean ± SEM of three independent experiments.

Furthermore, the growth pattern of unstressed wild-type and different mutants (*ΔendA*, *ΔcomEA*, and *ΔdprA*) was nearly identical to wild-type albeit the *ΔendA* mutant showed a slight slow growth pattern, indicating that gene (*ΔcomEA* and *ΔdprA*) deletions had no effect on the normal growth, while *ΔendA* gene deletion slightly slowed down the growth of *D. radiodurans* (Fig. 2A). However, when cells were exposed to a 6-kGy dose of gamma radiation, the *ΔendA* mutant exhibited some growth defects (Fig. 2B). Furthermore, the growth defect of the *ΔendA* mutant became more pronounced at a gamma dose of 12-kGy, showing a lag phase of approximately 600 minutes compared to 400 minutes for the wild-type (Fig. 2C). Interestingly, at a 12-kGy dose, the *ΔdprA* mutant showed slight fast growth compare to the wild-type (Fig. 2C). Together, the observation of long lag phase post-gamma irradiation indicated the defect in the DNA repair potential of the *ΔendA* mutant. For the *ΔdprA* mutant, enhanced growth at a higher gamma radiation dose (12-kGy) and reduced cell survival when DprA is constitutively expressed (Fig. 1C) are suggestive of a dominant negative effect of the *dprA* gene.

### Analysis of DSB repair profiles in *D. radiodurans* NT gene mutants

**Fig 3:**
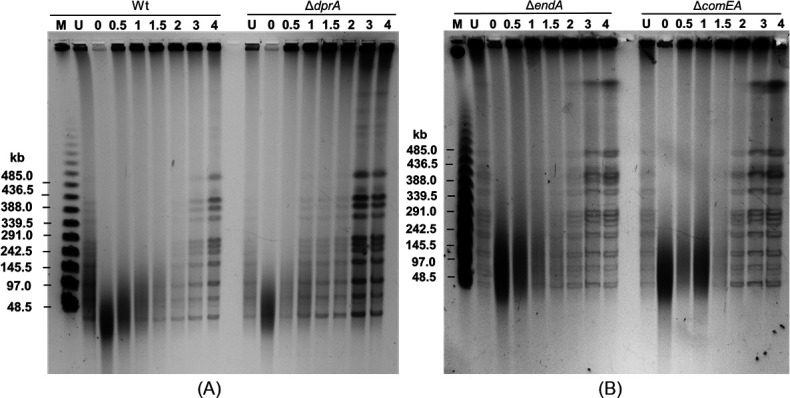
The kinetics of repair of DNA double-strand breaks (DSBs) in wild-type (Wt) and NT mutants of *D. radiodurans*. PFGE was utilized to evaluate the repair kinetics. The NotI-digested DNA from unirradiated cells (U) and irradiated cells at different post-irradiation time points after exposure to 6-kGy were visualized immediately after irradiation (0) and at specified incubation times (hours) on the gel. Lambda PFGE molecular mass standards (lane M). (**A**) The kinetics of DSB repair in Wt and *ΔdprA* mutants are shown. (**B**) The kinetics of DSB repair in *ΔendA* and *ΔcomEA* mutants. Data represented here are the representative of three independent experiments.

In order to investigate the DSB repair profiles of *ΔcomEA*, *ΔdprA*, and *ΔendA* mutants, pulsed-field gel electrophoresis (PFGE) was employed. The *ΔdprA* mutant exhibited faster DSBs repair kinetics following exposure to 6-kGy of gamma radiation, with distinct repaired genome bands appearing 1 hour post-irradiation (PIR) and repair nearly complete by 2 hours PIR (Fig. 3A, *ΔdprA*). In contrast, the wild-type strain showed delayed DSB repair compared to *ΔdprA*, with repaired genome bands becoming visible at 2 hours PIR and near completion repair by 4 hours PIR (Fig. 3A, wild-type). Similar result have been recently reported (24). The *ΔcomEA* and *ΔendA* mutants exhibited a DSB repair pattern nearly identical to that of the wild-type when exposed to 6-kGy gamma radiation (Fig. 3B). These results suggest that *comEA* and *endA* gene functions are not crucial for the DSB repair at 6-kGy gamma radiation dose or their functions are possibly compensated by functional homologs. This result is consistent with those observed in Fig. 1A. All together, these findings suggest that the *dprA* gene plays a direct role in DSB repair, and its absence leads to faster repair kinetics. Both the *ΔdprA* and *ΔendA* mutants were sensitive to higher doses of gamma radiation, though their sensitivity levels varied (Fig. 1A). However, their DSB repair kinetics differed; the *ΔendA* mutant had wild-type repair rates (Fig. 3B), while the *ΔdprA* mutant showed faster repair (Fig. 3A). This implies different gamma-induced DSB repair mechanisms in these mutants. Additionally, the PFGE banding pattern for NT-specific genes was similar to that of the wild-type, indicating no major genomic rearrangements (Fig. 3).

**Fig 4:**
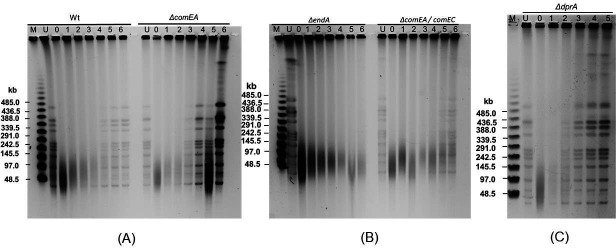
DNA double-strand break (DSB) repair kinetics in *D. radiodurans* wild-type and NT mutant strains treated with mitomycin C (MMC). The figure illustrates the kinetics of DSB repair in wild-type (Wt) and four NT-specific mutant strains of *D. radiodurans*—namely, *ΔcomEA*, *ΔendA*, *ΔcomEAΔcomEC*, and *ΔdprA*—following treatment with MMC. The repair kinetics were assessed using PFGE. NotI-digested DNA samples from untreated cells (U) and cells treated with 10-µg/mL MMC for 30 minutes were collected at various recovery time points (5–6 hours post-treatment) in TGY medium. These samples were analyzed by PFGE to evaluate the DSB repair process. Lane M contains molecular mass standards for reference. Data represented here are the representative of three independent experiments.

Unlike gamma radiation, the *ΔendA* mutant displayed significant sensitivity when treated with MMC (Fig. 1B). This sensitivity in the *ΔendA* mutant was due to defective DSB repair, as no detectable DSB repair was observed 6 hours post-MMC treatment (Fig. 4B). In contrast, the *ΔdprA* mutant demonstrated rapid repair of MMC-induced DSBs, with repair bands visible as early as 2 hours post-treatment and near-complete repair visible by 4 hours. This differs from the wild-type, where DSB repair began at 3 hours and took 6 hours to complete (Fig. 4C). Furthermore, the *ΔcomEA* mutant displayed a DSB repair profile similar to that of the wild-type, whereas the *ΔcomEA ΔcomEC* mutants showed little slower DSB repair (Fig. 4A and B). In summary, the findings indicate that in NT-specific gene mutants, gamma radiation-induced DSBs may exhibit wild-type-like repair kinetics in the *ΔcomEA* mutant, faster repair kinetics in the *ΔdprA* mutant, or slower/similar repair kinetics in the *ΔendA* mutant. In contrast, for MMC-induced DSBs, the repair kinetics are comparable to wild-type in the *ΔcomEA* and *ΔcomEA ΔcomEC* mutants but faster in the *ΔdprA* mutant and slower in the *ΔendA* mutant.

### *ΔendA* and *ΔdprA* single or *ΔendA ΔdprA* double mutants exhibited differential responses to DNA damage induced by combined gamma radiation + UV or MMC + UV treatments

Data from the preceding section suggested that the *ΔcomEA* and *ΔpilT* mutant strains did not show significant differences in cell survival compared to the wild-type strain under all tested conditions, indicating that these genes may not be necessary for *D. radiodurans* to cope with DNA damage (Fig. 1). The *ΔendA* mutant strain showed a decrease in survival to gamma radiation and MMC treatment, suggesting that the *endA* gene plays a role in coping with gamma radiation and MMC-induced DNA damage (Fig. 1, 3 and 4; see Fig. S1 at https://barc.gov.in/publications/aem01371-24r2/index.html). In contrast, the *ΔdprA* mutant strain showed susceptibility to gamma radiation at doses beyond 12-kGy and showed nearly similar survival after MMC treatment (Fig. 1; see Fig. S1 at https://barc.gov.in/publications/aem01371-24r2/index.html) albeit fast DBS repair kinetics (Fig. 3A and 4C), indicating that the *dprA* gene is crucial for coping with high doses of gamma radiation or MMC.

**Fig 5:**
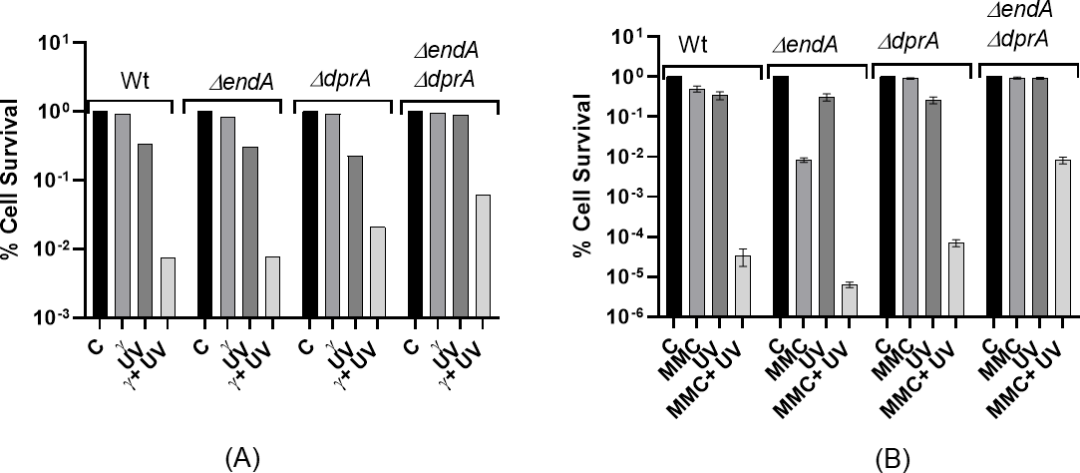
The cell survival of wild-type *D. radiodurans* and various NT mutants was measured after exposure to gamma radiation or MMC combined with UV radiation. The cell survival rates of wild-type *D. radiodurans* and various NT mutants were measured after exposure to gamma radiation or MMC combined with UV radiation. Exponentially growing cells of the *D. radiodurans* R1 wild-type and NT mutants were subjected to (**A**) 6-kGy doses of gamma radiation, 1,500-J/m² UV radiation, or both, and (**B**) MMC (20 µg/mL) for 30 minutes, 1,500-J/m² UV radiation, or both. The percentage of surviving cells was plotted for the different mutants. The mean ± SEM of three independent experiments (B) and a single data point representative of two independent experiments (A) are presented.

The contrasting responses of *ΔendA* and *ΔdprA* mutants to MMC and gamma radiation are intriguing, given that both treatments induce DSB and oxidative damages. The NT-specific EndA nuclease has been shown to support nutritional requirements under conditions of nutrient deprivation (104–106) or the dispersion of bacteria from biofilm (107–109). The decreased cell survival of *ΔendA* mutants against gamma radiation and MMC suggests that the EndA nuclease may support stressed cells in an unknown manner. We hypothesize that the *endA* gene might play a role in providing nutritional support to stressed *D. radiodurans* cells following exposure to gamma radiation or MMC. However, for eDNA to serve as a source of nutrition in the form of nucleosides, it must remain undamaged. To bolster this hypothesis, we treated wild-type and various mutants (*ΔendA*, *ΔdprA*, and *ΔendA ΔdprA*) with gamma radiation or MMC combined with UV radiation to induce complex DSB damage along with UV-induced cyclobutane pyrimidine dimers and 6–4 photoproducts. This combined treatment could severely damage cellular DNA, including eDNA, compromising its availability for nutrition. Our data indicate that gamma radiation alone (6-kGy) reduced the cell survival of the *ΔendA* mutant in a marginal reduction of 0.1-log cycle, while UV radiation (1,500 J/m²) resulted in a reduction of 0.3-log cycle (Fig. 5A; see Fig. S1 at https://barc.gov.in/publications/aem01371-24r2/index.html). The combined effect of gamma radiation and UV drastically reduced the cell survival of both the wild-type and *ΔendA* mutant, whereas for the *ΔdprA* mutant, gamma radiation alone (6-kGy) or UV radiation (1,500 J/m²) has an effect similar to *ΔendA* mutant (Fig. 5A), while the combined effect of gamma radiation and UV led to higher survival by approximately 0.6-log cycles compared to the wild-type and *ΔendA* mutant (Fig. 5A; see Fig. S1 at https://barc.gov.in/publications/aem01371-24r2/index.html). Conversely, the cell survival of *ΔdprA* mutants remained similar to that of the wild-type at selected MMC doses, yet the *ΔendA* mutant was nearly 2-log cycle sensitive in cell survival compared to wild-type (Fig. 5B). Furthermore, the combined effect of MMC and UV severely impacted the survival of the wild-type and *ΔendA* mutant (Fig. 5B). Interestingly, the *ΔdprA* mutant showed approximately 0.3-log cycle better survival to combined treatment of MMC and UV compared to the wild-type (Fig. 5B). This improved survival could be attributed to DprA role in the differential regulation of DSB repair pathways in *D. radiodurans*, as recently reported by us (24).

The *ΔendA* mutant, compromised in NT, and the absence of the *endA* gene led to reduced survival against gamma radiation, MMC, or combined gamma radiation + UV or MMC + UV treatment (Fig. 1 and 5; see Fig. S1 at https://barc.gov.in/publications/aem01371-24r2/index.html), highlighting the roles of *endA* in coping with genetic perturbations, possibly through its roles in nutritional support during stress (see Fig. S2 at https://barc.gov.in/publications/aem01371-24r2/index.html). Furthermore, in the absence of stress, the *ΔendA* mutant growth followed the wild-type pattern, suggesting that *endA* may not have roles in unstressed cells (see Fig. S2A at https://barc.gov.in/publications/aem01371-24r2/index.html). The *ΔdprA* mutant also showed a defect in NT, and its roles in dealing with gamma radiation and MMC-induced DNA damage are intriguing (Fig. 5). This reflects both its roles in NT and its differential effect on DSB repair pathways such as extended synthesis-dependent strand annealing (ESDSA) and SSA, as suggested recently (24). Interestingly, similar to the *ΔdprA* mutant, *ΔendA ΔdprA* double mutant showed significantly improved cell survival compared to the individual wild-type when treated with gamma radiation + UV or MMC + UV (Fig. 5A and B) and showed better DSB repair kinetics when treated with gamma radiation (see Fig. S3 at https://barc.gov.in/publications/aem01371-24r2/index.html). Additionally, this effect is supported by growth curve analysis of *ΔendA ΔdprA* double mutant treated with both gamma radiation and MMC (see Fig. S2D at https://barc.gov.in/publications/aem01371-24r2/index.html). The *ΔendA ΔdprA* double mutant showed the shortest lag phase and started to recover faster than the wild-type or individual mutants of *endA* and *dprA* genes (see Fig. S2D at https://barc.gov.in/publications/aem01371-24r2/index.html), corroborating the findings of Fig. 5. Again, this improved survival could be attributed to DprA role in the differential regulation of DSB repair pathways in *D. radiodurans* (24). Thus, the hypothesis that severely damaged eDNA is less utilized for nutritional purposes is strongly supported by data showing a severe reduction in cell survival of the *ΔendA* mutant when gamma radiation or MMC treatment is combined with UV radiation (Fig. 5). Additionally, our findings support previous observations that *D. radiodurans* continuously acquires eDNA during its growth. It can repair UV radiation-damaged transforming DNA through its highly effective repair system, even after chromosomal conversion (94, 110).

### NT-specific genes (*endA*, *comEA*, and *comEC*) required for the DNA as a nutritional source

Bacteria can utilize DNA as a source of phosphorus, carbon, and nitrogen for growth (104, 111–114). This is achieved by internalizing eDNA into their periplasm, where periplasmic DNases and phosphatases process it for nutritional or structural purposes (104–106, 109, 111, 115). The ComEA protein, which is a receiver of transforming DNA in the periplasm, is localized in the periplasm of *D. radiodurans* with high reliability. Bioinformatics analysis using the DeepTMHMM server (DeepTMHMM-1.0) (https://services.healthtech.dtu.dk/service.php?DeepTMHMM-1.0) predicted that ComEA contains a transmembrane helix at its N-terminus (residues 7–29), with the remainder of the protein (residues 30–130) located in the periplasmic region. In contrast, *D. radiodurans* EndA is predicted to be cytoplasmic and lacks any signal peptide, as analyzed using the SignalP 6.0 server (https://dtu.biolib.com/SignalP-6). The *D. radiodurans* EndA catalytic core showed substantial sequence similarity with approximately 25% sequence identity and 30% sequence similarity to *Escherichia coli*, *V. cholerae*, and *Aeromonas hydrophila* EndA (see Fig. S4 at https://barc.gov.in/publications/aem01371-24r2/index.html). However, the N-terminal region (residues 1–90) of *D. radiodurans* EndA shows little sequence identity compared to other EndA proteins (see Fig. S4 at https://barc.gov.in/publications/aem01371-24r2/index.html). Nonetheless, *D. radiodurans* has a protein transport system to send proteins across the cytoplasmic membrane via the SecA pathway or the signal recognition particle (SRP) pathway, and it is shown that many designated cytoplasmic proteins, including catalases (KatE1 and KatE2), are transported to the periplasm using SRP pathways (116). EndA, which is a designated periplasmic protein in many bacteria including *E. coli* (104), *V. cholerae* (96, 117), and *Streptococcus pneumoniae* (118, 119), could have a signal peptide for periplasmic location or could be transported to the periplasm by various transport systems such as Tat, SecA, or SRP pathways (120, 121). Since EndA function is crucial for *D. radiodurans* survival under gamma radiation and MMC stress (Fig. 1 and 5), it likely enables the use of eDNA from lysed or secreted cells for nutrition or DNA repair. This eDNA can be transported into the periplasmic space of *D. radiodurans*, where the action of EndA liberates free nucleotides as well as ssDNA from incoming dsDNA. Both free nucleosides or nucleosides and ssDNA (once entered inside the cell) can be further utilized as nutrition sources of phosphorus (P), carbon (C), and nitrogen (N).

**Fig 6:**
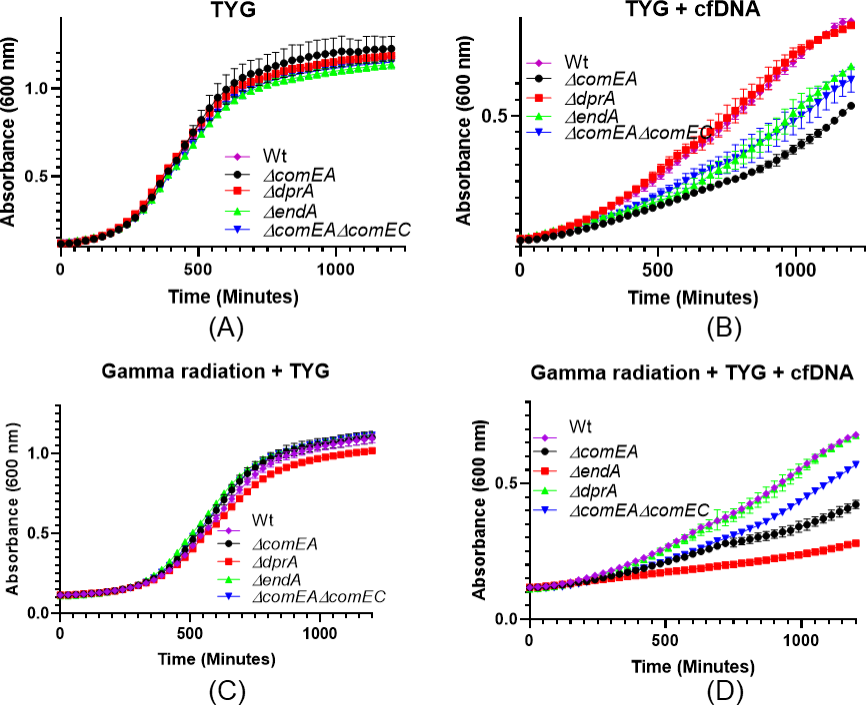
Cell growth curve of wild-type and NT mutants after exposure to calf thymus DNA (cfDNA) in TGY growth medium with and without gamma radiation. Optical density at 600 nm was continuously measured using a microtiter-based density reader. (**A**) Growth of normal, untreated cells in the TGY growth medium. (**B**) Cells treated with cfDNA (0.5 mg/mL) in a TGY medium. (**C**) Cells treated with a dose of 6-kGy of gamma radiation in a TGY medium. (**D**) Cells treated with a dose of 6-kGy of gamma radiation in a TGY medium supplemented with cfDNA. The data presented here are the mean ± SEM from three independent experiments.

We have attempted to test this hypothesis by growing the *D. radiodurans* in the minimal medium where calf thymus DNA (cfDNA) is used as the sole C or N source (see Fig. S5 at https://barc.gov.in/publications/aem01371-24r2/index.html). Unfortunately, poor growth support of *D. radiodurans* in minimal medium with and without cfDNA (0.5 mg/mL) added was observed (see Fig. S5 at https://barc.gov.in/publications/aem01371-24r2/index.html). To further substantiate the findings, we have grown wild-type as well as different mutants (*ΔendA*, *ΔdprA*, *ΔcomEA*, and *ΔcomEA ΔcomEC*) in TGY medium or TGY medium supplemented with cfDNA DNA (0.5 mg/mL). Data suggested that in TGY medium, wild-type and all mutants grow nearly identically and follow the standard growth trajectories (Fig. 6A). However, in the presence of cfDNA *ΔendA*, *ΔcomEA*, *and ΔcomEA ΔcomEC* mutants, growth slowed down compared to *ΔdprA* and wild-type (Fig. 6B). Interestingly, it is observed that growth of the wild-type cells and other mutants is substantially slowed down in the TGY + cfDNA medium. For example, wild-type required ~400 minutes to reach 0.5 optical density (OD) in TGY medium, which increased up to ~750 minutes in the TGY + cfDNA medium to reach similar OD values (compare Fig. 6A and B). The observed doubling in the time required to reach the exponential phase in the presence of cfDNA suggests that utilizing DNA as a nutritional source may take more time. However, this effect was absent when the cfDNA was pre-digested with DNase and added to the TGY medium (see Fig. S6 at https://barc.gov.in/publications/aem01371-24r2/index.html). Instead, *D. radiodurans* cells showed improved growth when supplied with pre-digested cfDNA in the TGY medium, regardless of gamma radiation treatment (6-kGy). In contrast, undigested cfDNA inhibited the growth of both irradiated and non-irradiated cells (see Fig. S6 at https://barc.gov.in/publications/aem01371-24r2/index.html). This finding indicates that small DNA fragments or nucleosides can be readily utilized by *D. radiodurans* cells. This notion is further supported by growth data obtained for *ΔendA*, *ΔcomEA*, and *ΔcomEA ΔcomEC* mutants, whether in unstressed conditions (Fig. 6B) or gamma radiation-stressed cells supplemented with cfDNA (Fig. 6D). It was observed that these mutants, which are defective in receiving eDNA in the periplasm (*ΔcomEA*) or defective in both receiving DNA in the periplasm and transporting it through the inner membrane (*ΔcomEA ΔcomEC*), or defective in converting dsDNA to ssDNA in the periplasm (*ΔendA*), exhibited delayed growth (Fig. 6B and D). This delay is likely due to their inability to utilize DNA as a nutritional source under both unstressed and stressed conditions. Together, these data suggested that *D. radiodurans* grows better when small DNA or nucleotides or nucleosides are available in a growth medium in both stressed and unstressed conditions. Furthermore, mutants lacking specific DNA-processing proteins exhibit delayed growth. This suggests that internal processing of eDNA in wild-type is more efficient and advantageous for bacterial survival and growth.

## DISCUSSION

The results of this study provide insights into the role of NT-specific genes (*ΔcomEA*, *ΔpilT*, *ΔendA*, and *ΔdprA*) in the survival and DNA repair mechanisms of *D. radiodurans* under various stress conditions, including exposure to gamma radiation, MMC, and combinations of gamma radiation or MMC with UV radiation. The first key finding is the differential cell survival response observed among the NT-specific gene mutants when exposed to gamma radiation and MMC. While *ΔcomEA* and *ΔpilT* mutants exhibited survival rates similar to the wild-type, the *ΔendA* and *ΔdprA* mutants displayed decreased survival under specific stress conditions (Fig. 1; see Fig. S1 at https://barc.gov.in/publications/aem01371-24r2/index.html). Although the ComEA function is crucial for natural transformation in many bacteria, in *D. radiodurans*, it is likely that in the absence of ComEA (DR1854), its functional homolog, the ComEA-like protein (DR0207), may compensate for its loss. Notably, *ΔendA* and *ΔdprA* mutants showed increasing susceptibility to higher doses of gamma radiation (Fig. 1A), while the *ΔendA* mutant showed a significant decrease in survival upon exposure to MMC, indicating a potential role for the *endA* gene in coping with MMC-induced DNA damage (Fig. 1B). Additionally, the growth patterns of the mutants were comparable to that of the wild-type under normal conditions, but defects were observed post-gamma irradiation or MMC treatment, particularly for the *ΔendA* (slow growth) and *ΔdprA* (fast growth), suggesting impaired DNA repair potential (Fig. 2; see Fig. S2 at https://barc.gov.in/publications/aem01371-24r2/index.html). Studies found that both gamma radiation and MMC primarily cause DSBs to the genome, but the mechanisms of DSB generation differ between the two agents. Additionally, gamma radiation introduces oxidative stress and ssDNA breaks, while MMC also damages proteins and RNA (122–124). The gamma radiation causes the DSBs by direct deposition of energy to phosphodiester bonds or indirectly through reactive oxygen species and other radicals. MMC causes the inter- and intra-strand cross-links in DNA, and these cross-links seldom converted to DSBs through DNA replication (125, 126). Thus, it could be conceivable that the mechanism of biomolecule damage and DSB generation is different for gamma radiation and MMC (58, 123, 127, 128). Thus, it is plausible that DSB repair kinetics in NT mutants may follow differently when treated with gamma radiation and MMC. The *ΔdprA* mutant exhibited faster DSB repair kinetics post-gamma irradiation (Fig. 3A), whereas the *ΔendA* mutant showed slower repair compared to the wild-type (Fig. 3B). These findings suggest that DprA and EndA are involved in DSB repair, with their absence resulting in altered DSB repair kinetics (Fig. 3). Interestingly, the fast DSB repair as exhibited by the *ΔdprA* mutant and the slow DSB repair as shown by the *ΔendA* mutant, indicating distinct DSB repair mechanisms for different types of DNA damage and mutant background, highlight the complexity of repair followed in *D. radiodurans* (Fig. 3 and 4). Additionally, the observation that the *ΔendA* mutant growth was affected by gamma radiation (Fig. 2C) and MMC (see Fig. S2C at https://barc.gov.in/publications/aem01371-24r2/index.html), resulting in a prolonged lag phase, further supports the notion that EndA plays a role in DNA repair and that its absence leads to a defect in the ability of *D. radiodurans* to recover from DNA damage.

Under specific conditions, bacteria have shown the ability to utilize DNA as elemental sources of P, C, and N for their growth (104, 111–114). This is achieved through two mechanisms. (i) Bacteria secrete DNase into the environment, where these nucleases degrade the free-form eDNA into nucleotides or nucleosides, which are then utilized by bacteria (112, 129). (ii) Bacteria also utilize secretory phosphatases to release phosphate from eDNA for subsequent utilization. However, secreting DNase/phosphatases into the environment is not advantageous because bacteria typically coexist with many other bacterial species. The digested DNA monomers and fragments are easily taken up by competing bacteria, and cross-feeding reduces the survival benefits. Furthermore, the effective concentration of DNase becomes much lower if it is secreted into the culture medium, making it energetically unfavorable for the bacteria. Thus, bacteria adopt another effective, energy-efficient, and competition-free mechanism, which is to acquire eDNA into the bacterial periplasm and then utilize it for nutrition or as a structural constituent in biofilms with the help of periplasmic DNase and phosphatases (104–106, 109, 111, 115). The NT-specific EndA nuclease has been demonstrated to aid in nutritional needs during nutrient deprivation (104–106) and in the dispersion of bacteria from biofilms (107–109). The reduced cell survival observed in *ΔendA* mutants exposed to gamma radiation and MMC indicates that the EndA nuclease might assist stressed *D. radiodurans* cells, possibly by providing nutritional support after exposure to gamma radiation or MMC. The combined effects of gamma radiation and UV or MMC and UV on cell survival were evaluated with the assumption that combined treatment could severely damage cellular DNA, including eDNA, thereby compromising its availability for nutrition. The *ΔendA* and *ΔdprA* mutants exhibited different responses to these treatments, with the *ΔdprA* mutant showing improved survival when gamma radiation or MMC was combined with UV (Fig. 5). The *Δend* mutant and the *ΔendA ΔdprA* double mutant, both of which lack the ability to undergo natural transformation, exhibited distinct responses. The *Δend* mutant demonstrated reduced survival, whereas the *ΔendA ΔdprA* double mutant showed improved survival and faster DSB repair kinetics and growth when treated with gamma radiation + UV or MMC + UV (Fig. 5; see Fig. S2D and S3 at https://barc.gov.in/publications/aem01371-24r2/index.html). In wild-type cells, both the SSA and ESDSA pathways contribute to DNA repair. However, in the absence of DprA, SSA becomes the dominant pathway, leading to a reduction in ESDSA efficiency (24). The decision between SSA and ESDSA depends on factors such as the length of the ssDNA tail and the presence of homologous sequences. In *D. radiodurans*, the balance between these pathways is crucial for survival under genotoxic stress (31). SSA plays a role in initiating ESDSA by generating the appropriate substrate, while RecA, essential for ESDSA, searches for homology to enable error-free repair (97). *D. radiodurans* cells subjected to high levels of gamma irradiation, small DNA fragments may limit the function of RecA, making the RecA-independent phase critical for efficient DSB repair. These findings suggest that DprA, through its RMP-like (RecA mediator protein-like) activity, may effectively compete with repair-specific RMPs like RecFOR for loading RecA onto SSB (single-strand binding protein)-coated ssDNA ends, thereby slowing down the ESDSA repair process and indirectly enhancing the preference for SSA repair (24). In *D. radiodurans*, the level of RecA increases immediately after exposure to acute doses of gamma radiation. However, RecA role in ESDSA and HR-mediated repair becomes significant only after a delay of approximately 1.5 hours during post-irradiation recovery (33, 34). Therefore, it is crucial to regulate RecA function until SSA repair is active in the early hours of post-irradiation recovery. Moreover, DprA interference with RecA recombination activity appears to be an effective mechanism for controlling RecA activity and efficiently utilizing different DSB repair pathways (24). Thus, DprA protein RMP-like activities justify its inhibitory effect on ESDSA repair. The role of DprA in the differential regulation of SSA and ESDSA repair in *D. radiodurans* also suggests an intriguing interaction between DprA and EndA during post-irradiation recovery. The observed increased resistance of *ΔdprA* and *ΔendA ΔdprA* mutants to various stress factors could be due to DprA protein roles in DSB repair as well as its involvement in NT. This effect is more pronounced in the *ΔendA ΔdprA* double mutant compared to the *ΔdprA* single mutant, possibly because DprA functions are divided between NT and DSB repair. The *ΔdprA* mutant is partially deficient in NT, while the *ΔendA* and *ΔendA ΔdprA* double mutant is completely deficient in NT. Collectively, these results support the hypothesis that severely damaged eDNA is less utilized for nutritional purposes, while the possible differential inhibitory effect of DprA on DSB repair pathways leads to elevated cell survival and growth under stress from gamma radiation, MMC, or both agents. We further substantiate the notion that DNA can be utilized as a nutritional resource under stressed conditions, with EndA and ComEA potentially playing roles in this process within the periplasmic space. Our analysis of the growth patterns of the *ΔendA*, *ΔcomEA*, and *ΔcomEA ΔcomEC* mutants in the presence of cfDNA reveals that these mutants are impaired in their ability to utilize DNA as a nutritional source in gamma radiation-stressed conditions and unstressed conditions, likely due to their inability to process eDNA effectively (Fig. 6).

**Fig 7:**
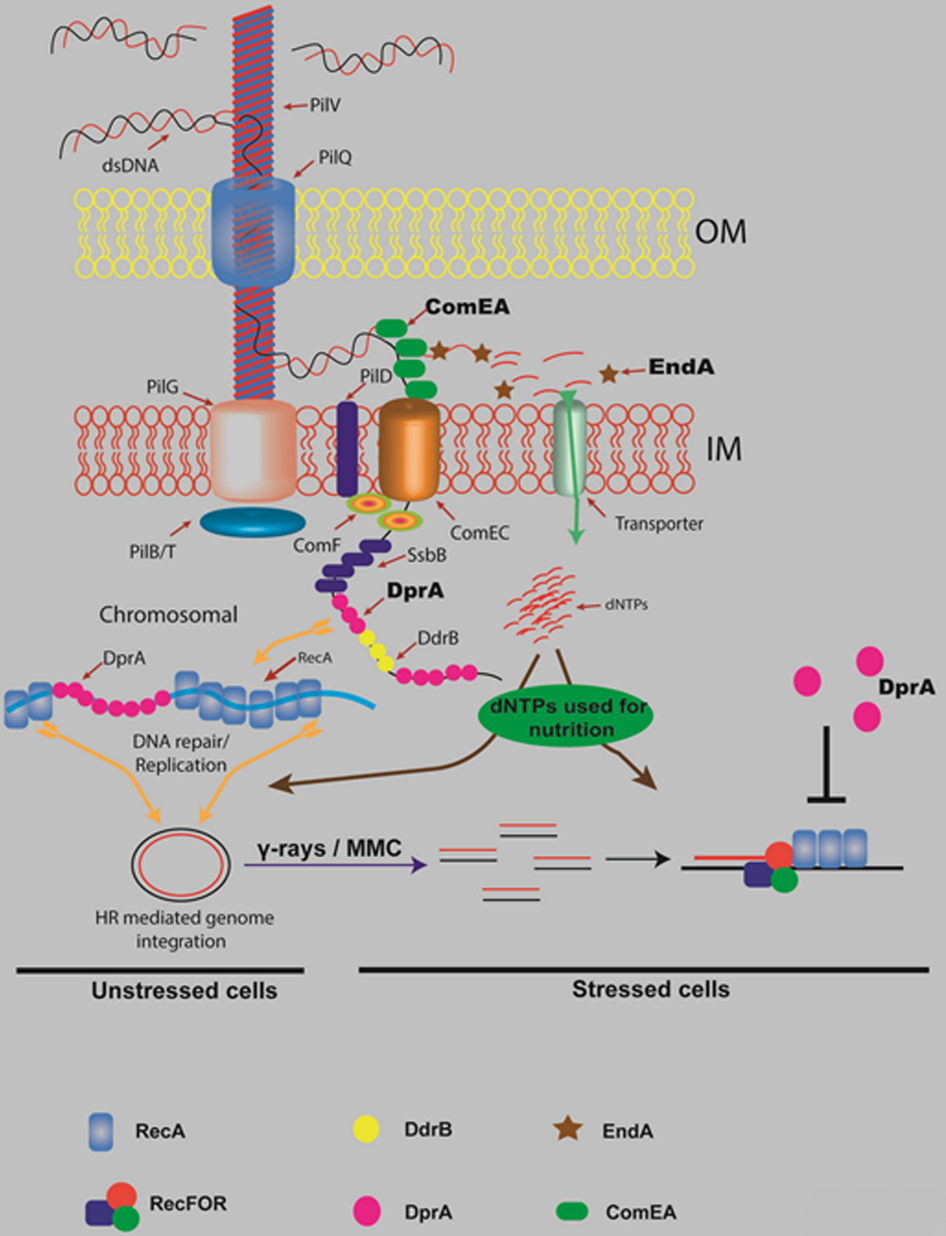
Model of DNA uptake apparatus and DNA processing during natural transformation in *D. radiodurans*, highlighting roles of ComEA, EndA, and DprA proteins. This study, along with other published research, proposes a model of natural transformation in *D. radiodurans*. Extracellular DNA binds to the type IV pilus-like DNA uptake machinery, which includes homologs of the pilins PilIV, the outer membrane (OM) channel PilQ, the ATPases PilB and PilT, and the pre-pilin peptidase PilD. Once bound, double-stranded DNA (dsDNA) passes through the OM via the PilQ channel, where it is received in the periplasm by the DNA-binding protein ComEA (DR1855) or a ComEA-like protein (DR0207). In the periplasm, EndA (DR1600) degrades one strand of the dsDNA, producing single-stranded DNA (ssDNA) for translocation into the cytoplasm through the inner membrane (IM) channel ComEC (DR1854), possibly with assistance from ComF. The degraded DNA strand produces small DNA fragments or nucleosides, which may be transported into the cytoplasm via putative transporters for small DNA fragments or nucleosides, contributing to the nutritional needs of both unstressed and stressed cells. After entering the cytoplasm, intact ssDNA is protected by single-stranded DNA-binding proteins, such as SSB, DdrB, and DprA (DR0120). DprA, bound to the incoming DNA, facilitates the loading of RecA on it and promote homologous recombination with the host cell genome. Additionally, DprA may play a crucial role in selecting between single-strand annealing and extended synthesis-dependent strand annealing DSB repair pathways in stressed cells. This may occur through DprA ability to compete with the RecFOR complex for the ssDNA repair intermediates.

Together, the findings from this study have significant implications for our understanding of natural transformation roles in DNA repair and cell survival mechanisms in *D. radiodurans*. The findings of this study, along with other published research, are summarized in the model presented in Fig. 7. The differential responses of the *ΔendA* and *ΔdprA* mutants to DNA-damaging agents highlight the complexity of the DNA repair processes in this organism. The observation that the *ΔdprA* mutant exhibits faster DSB repair kinetics is particularly noteworthy. This finding suggests that DprA may be involved in NT as well as the regulation of DSB repair pathways of *D. radiodurans* (Fig. 7). The role of EndA in the *D. radiodurans* response to DNA damage is also of great interest. The slower DSB repair kinetics observed in the *ΔendA* mutant and the growth defects following gamma radiation/MMC exposure suggest that EndA plays a critical role in the repair of DSBs. The hypothesis that EndA supports stressed cells through nutritional means is strengthened by the data provided in this study (Fig. 7). However, further research is needed to elucidate the exact mechanisms by which EndA contributes to cell survival under stress by offering nutritional support. Additionally, the roles of DprA suggested that relay on eDNA for DNA repair may have detrimental or inconclusive consequences as shown for other bacteria (9, 15, 22, 104, 107, 111). Thus, the ability of *D. radiodurans* to utilize eDNA for nutrition or DNA repair, whether under unstressed or stressed conditions, likely involves complex dynamics and consequences and repercussions.

## MATERIALS AND METHODS

### Bacterial strains and plasmids, chemicals, and growth medium

In this investigation, a range of bacterial strains were utilized, including the wild-type *D. radiodurans* R1 (ATCC 13939) sourced from the ATCC. The construction of *ΔcomEA*, *ΔdprA*, *ΔendA*, *ΔdprA ΔendA*, and *ΔpilT* mutants was performed in our laboratory using previously described methods (48). Additionally, *ΔcomEA ΔcomEC* double mutants were obtained from Prof. Pascale Servant, Université Paris-Sud Orsay, France. All strains were cultured in TGY medium (1% Bacto tryptone, 0.1% glucose, and 0.5% yeast extract) with the addition of suitable antibiotics, following the established protocol (87). The media were supplemented with various antibiotics, specifically kanamycin (8 µg/mL), chloramphenicol (3 µg/mL), and tetracycline (2 µg/mL) for *D. radiodurans* and ampicillin (100 µg/mL) for *E. coli*. The shuttle vector p11559 was maintained using spectinomycin at concentrations of 70 µg/mL for *D. radiodurans* and 150 µg/mL for *E. coli*. Additionally, the shuttle expression vector pRADgro and its derivatives were maintained in the *E. coli* strain HB101, as previously described (35). Standard molecular biology techniques were employed as described in *Molecular Cloning: a Laboratory Manual* (130). Molecular biology-grade chemicals, enzymes, and salts were sourced from Sigma Chemicals Company (USA), Roche Biochemicals (Mannheim, Germany), New England Biolabs (USA), and Merck India Pvt. Ltd. (India). For a comprehensive list of the bacterial strains and plasmids used in this study, please refer to Table S1.

### Cell survival studies

The experimental treatments for studying the survival of *D. radiodurans* cells involved exposure to various doses of UV and gamma radiation, as previously detailed (83), as well as treatment with MMC (10 µg/mL) according to the protocol described in a previous study (87). Briefly, bacterial cultures grown in TGY medium at 32°C were washed and suspended in sterile phosphate-buffered saline. These cultures were then exposed to varying doses of gamma radiation at a dose rate of 5.86-kGy/hour using a Gamma 5000 (^60^Cobalt) irradiator from the Board of Radiation and Isotope Technology, DAE, India. For UVC treatment, different dilutions of the cells were plated and subjected to 1,500-J/m^2^ doses of UV radiation at 254 nm. The treated cells were subsequently plated on TGY agar plates, supplemented with appropriate antibiotics if necessary, and the colony-forming units were counted after a 48-hour incubation period at 32°C. For cells receiving both gamma radiation and MMC treatments, the protocol involves administering gamma radiation first, followed by MMC treatment. When combining UV with either gamma radiation or MMC, the sequence begins with gamma radiation or MMC treatment followed by UV exposure. For the spot assay, wild-type and NT gene mutants of *D. radiodurans* were treated with appropriate DNA-damaging agents alone or in combination with UV radiation cells diluted to appropriate serial dilution and 5 µL spotted on TGY plates supplemented with appropriate antibiotic wherever needed.

### Measurement of DNA repair kinetics using PFGE

Irradiated cultures were diluted in TGY to an OD_600_ of 0.2 and incubated at 32°C. At specified intervals, 5-mL samples were collected to prepare DNA plugs as described by Mattimore and Battista (131). The DNA in the plugs was digested with 60 units of NotI restriction enzyme (Roche) for 16 hours at 37°C. Following digestion, the plugs underwent pulsed-field gel electrophoresis in 0.5× Tris-borate-EDTA (TBE) buffer using a CHEF-DR III electrophoresis system (Bio-Rad) at 6 V/cm² for 20 hours at 14°C, with a linear pulse ramp of 50–90 seconds and a switching angle of 120°.

### Growth curve studies

*D. radiodurans* cells were cultured in TYG broth (containing 0.5% tryptone, 0.3% yeast extract, and 0.1% glucose) at 32°C under atmospheric conditions. The cultures were grown in a 24-well, energy-treated microplate (Nunc, Thermo Scientific) for 16–20 hours using a BioTek Microplate Reader (Agilent Technologies, USA). For experiments requiring double-stranded cfDNA, a solution of 10-mg/mL deoxyribonucleic acid sodium salt from cfDNA (Sigma-D1501) was prepared and sonicated under sterile conditions. A final concentration of 0.5-mg/mL DNA was then added to either the minimal medium (132) or the TYG medium as needed. The composition of the minimal medium for *D. radiodurans* was adopted from previously published sources (132).

### Statistical methods

The data presented in the figures represent the average values from at least three independent experiments, with the results shown as the mean ± SEM or the most representative of the three independent experiments. For Fig. 5A and S3 (https://barc.gov.in/publications/aem01371-24r2/index.html), the data were obtained from two independent experiments.

## Data Availability

The authors confirm that the data supporting the findings of this study are available within the article or its supplemental materials (https://barc.gov.in/publications/aem01371-24r2/index.html). However, specific data that support the findings of this study are available from the corresponding author (email: ysraj@barc.gov.in) upon request.
